# Estimated Cases Averted by COVID-19 Digital Exposure Notification, Pennsylvania, USA, November 8, 2020–January 2, 2021

**DOI:** 10.3201/eid2902.220959

**Published:** 2023-02

**Authors:** Seonghye Jeon, Gabriel Rainisch, A-Mac Harris, Jared Shinabery, Muneeza Iqbal, Amar Pallavaram, Stacy Hilton, Saugat Karki, Patrick K. Moonan, John E. Oeltmann, Martin I. Meltzer

**Affiliations:** Centers for Disease Control and Prevention, Atlanta, Georgia, USA (S. Jeon, G. Rainisch, S. Karki, P.K. Moonan, J.E Oeltmann, M.I. Meltzer);; Pennsylvania Department of Health, Harrisburg, Pennsylvania, USA (A.-M. Harris, J. Shinabery, M. Iqbal, A. Pallavaram, S. Hilton)

**Keywords:** COVID-19, respiratory infections, severe acute respiratory syndrome coronavirus 2, SARS-CoV-2, SARS, coronavirus disease, zoonoses, viruses, coronavirus, digital exposure notification, cases averted, Pennsylvania, United States

## Abstract

We combined field-based data with mathematical modeling to estimate the effectiveness of smartphone-enabled COVID-19 exposure notification in Pennsylvania, USA. We estimated that digital notifications potentially averted 7–69 cases/1,000 notifications during November 8, 2020–January 2, 2021. Greater use and increased compliance could increase the effectiveness of digital notifications.

Case investigation and contact tracing (CICT) was a pillar among COVID-19 prevention strategies, especially before vaccine availability (*1*,*2*). However, standard CICT relies on staff to reach cases and close contacts, which is labor intensive, and CICT programs often become overwhelmed when caseloads surge (*3*–*5*). Standard CICT also relies on case investigation interviews to identify contacts; thus, it is prone to recall and participation bias and might not identify all potential exposures, such as interactions between strangers in public spaces.

COVID-19 exposure notification smartphone applications (apps) can alleviate those challenges by automatically notifying app users when they have been near other users who reported positive SARS-CoV-2 results (herein referred to as cases). Pennsylvania, USA, and 26 other states implemented digital exposure notifications to complement their standard CICT programs (*6*). However, few studies have evaluated the effectiveness of digital notifications in the United States (*6*,*7*). 

We estimated the number of cases and hospitalizations averted by Pennsylvania’s digital notification system, COVID Alert PA app. We also investigated strategies to increase the system’s efficiency and its effects on the estimated number of cases and hospitalizations. 

## The Study

During case investigation interviews in Pennsylvania, digital notification app users were identified and given a validation code to enter into their app. The app then automatically sent anonymous notifications to other users identified through smartphone Bluetooth technology as potentially exposed to the person testing positive for COVID-19 (Appendix).

The Pennsylvania Department of Health (PA DoH) collected data on the performance of standard CICT and digital notification apps (Table). We aggregated those data across all counties, excluding Philadelphia County (Appendix), for 8 weeks, November 8, 2020–January 2, 2021 (Table). We extracted the daily number of COVID-19 cases from the Centers for Disease Control and Prevention (CDC) COVID Data Tracker (*8*).

**Table:** Reported and estimated program metrics in a study of estimated cases averted by COVID-19 digital exposure notification, Pennsylvania, USA, November 8, 2020–January 2, 2021*

Program	Reported metrics
Standard case investigation and contact tracing	
Cases interviewed, no. (% total cases)	77,477 (20)
Cases named >1 contact, no. (% interviewed cases)	32,648 (42)
No. contacts named	48,615
Contacts notified and monitored, no. (% identified contacts)	26,203 (54)
Contacts notified but not monitored, no. (% identified contacts)	418 (1)
Timing of case interview, d†	5
Timing of contact notification, d‡	6
Digital exposure notification	
Median no. active daily users (% total population)§	356,835 (3.2)
Cases interviewed and identified as app user, no. (% total cases)	786 (0.2)
No. validation codes generated (% cases that had the app installed)	579 (74)
No. validation codes claimed and certified (% cases that had the app installed)	390 (50)
Timing of digital notification, d‡	6
Estimated program effectiveness#	
Cases and contacts isolated or quarantined, %**	7–11.7
Days from infection to isolation or quarantine	10

*Data excludes Philadelphia County. CICT, case investigation and contact tracing. †Reported average number of days from specimen collection to case interview. ‡Reported average number of days from specimen collection to contact notification. §For Android users, the total number of devices that were turned on >1 time in the past 30 d. For iOS users, the total number of devices with >1 session within 30 d of the selected day. During the study period, only persons >18 years of age were eligible to download and activate the digital notification application on their smartphone devices; thus, data provided is equivalent to 4.0% of the eligible population. #Calculations provided in the Appendix . **Includes contacts that later become cases. The range reflects the lowest and highest values across 18 studied scenarios of compliance with quarantine guidelines and the degree of overlap between notifications received via the COVID Alert PA app and by Pennsylvania Department of Health staff members (Appendix Tables 4, 5). The low-value results from a scenario assuming 50% of digital notifications were sent to contacts that were already notified by Department of Health staff members and 10% of notified contacts followed quarantine guidance. The high-value results from a scenario assuming all digital notifications were sent to contacts that were not notified via standard CICT and 50% of notified contacts followed quarantine guidance.

We used CDC’s COVIDTracer modeling tool to estimate cases and hospitalizations averted by digital notifications during the 8-week study period (*1*,*2*,*9*). COVIDTracer uses an epidemiologic model to illustrate the spread of COVID-19 and effects of CICT and other nonpharmaceutical interventions (NPIs). We calculated a summary effectiveness measure for CICT and digital notification apps from the various data PA DoH collected and input this measure to the model (Table). We defined this summary effectiveness measure as the proportion of cases that entered isolation and contacts that quarantined in response to CICT and digital notification apps, and the number of days required to do so (i.e., number of days from exposure to isolation or quarantine). We further assumed 60%–100% of interviewed cases and monitored contacts fully adhered to isolation and quarantine guidelines, and that 10%–50% of notified but not monitored contacts complied with quarantine guidance (*10*–*12*). To calculate the number of days from exposure to isolation or quarantine, we averaged the number of days between case interviews (triggering case isolation) and contact notifications (triggering contact quarantine). We performed 2 sensitivity analyses by varying the estimated number of days from infection to isolation by +1 day and the weight used to estimate the overall proportion of cases isolated and contacts quarantined (Appendix).

We derived CICT program effectiveness from reported data, but data were not available to estimate effectiveness of other NPIs, such as social distancing and mask-wearing. Therefore, we used the tool to estimate the effectiveness of other NPIs by fitting the model-generated curve to observed case curve (Appendix). Finally, to show what might have happen without the digital notifications, we simulated a hypothetical case curve by replacing the CICT effectiveness input with a value excluding contributions of the digital notifications. We considered the difference between cases in the simulated curve and reported cases as the estimated cases averted by the digital notifications. We generated a range of 18 results by varying public compliance with isolation and quarantine guidance and the degree to which recipients of digital notifications were also notified by the PA DoH staff members. First, we assumed no overlap (i.e., all digital notifications were sent to contacts who were not notified by the DoH staff); then, we assumed a 50% overlap (Appendix Tables 4, 5). We also calculated the number of hospitalizations averted by multiplying the estimated number of averted cases by age-stratified infection-to-hospitalization rates (*9*). We did not account for vaccination because only 0.1% of Pennsylvania’s population was fully vaccinated during the study period.

Between its launch in late September and the end of the study period, Pennsylvania’s digital notification app was downloaded 638,797 times, accounting for ≈5.7% of the population; 56% (n = 356,835) of downloaded apps were actively used, accounting for 3.2% of the population. In all, 786 interviewed case-patients (0.2% of all cases) had the digital notification app installed on their smartphones, among whom <50% (n = 390) used the app to notify others of potential exposure, totaling 233 digital notifications during the 8-week period (Table).

**Figure 1:**
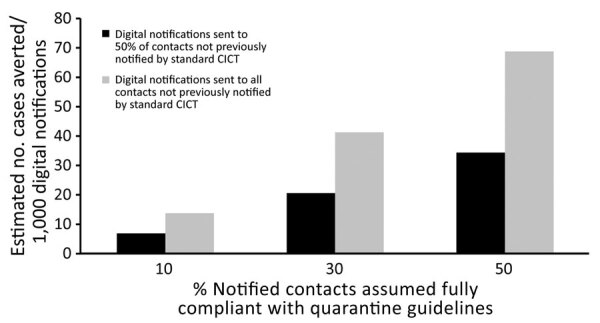
Estimated number of cases averted per 1,000 COVID-19 digital notifications, Pennsylvania, USA, November 8, 2020–January 2, 2021. Estimates show selected scenarios of isolation or quarantine compliance and the digital notification application’s ability to identify previously unknown contacts. Data from Philadelphia County are excluded. The figure represents a scenario in which 80% of interviewed cases and monitored contacts comply with isolation and quarantine guidance. We also modeled 60% and 100% compliance scenarios (Appendix Tables 4, 5). At just 10% compliance among notified contacts, digital notifications averted 7 cases/1,000 notifications (or 2 cases); at 50% compliance among notified contacts, digital notifications averted 69 cases/1,000 notifications (or 16 cases). CICT, case investigation and contact tracing.

We estimated those digital notifications averted 2–16 additional cases (7–69 cases/1,000 notifications) and <1 hospitalization (Figure 1; Appendix Tables 4, 5). That range reflects uncertainties in both public compliance and the degree of overlap between notifications received via the digital notification app and DoH staff. In comparison, we estimated standard CICT averted 10,168–17,151 cases and 250–421 hospitalizations during the same period.

## Conclusions

Although just 3.2% of the state’s population used the COVID Alert PA app, we estimated that 7–69 cases were averted for every 1,000 digital notifications sent during the 8-week study. Those estimates represent a single locality and should not be generalized to other jurisdictions. However, the methods, and the publicly accessible modeling tool, could be used to adjust for differences in uptake, compliance, and epidemic curve to estimate the effect of digital notifications in other jurisdictions.

**Figure 2:**
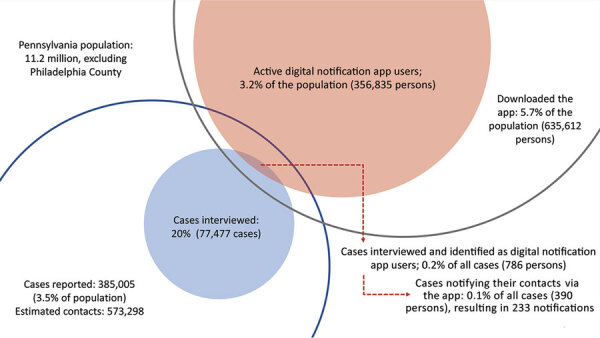
Overlap between standard CICT and digital notifications in a study of estimated cases averted by COVID-19 digital exposure notification, Pennsylvania, USA, November 8, 2020–January 2, 2021. During the study period, standard CICT resulted in interviews and contact elicitation from 20% of the reported cases (blue, shaded circle) and 3.2% of the population actively used the digital notification app (red, shaded circle). During case interviews, app users were provided validation codes for initiating contact notifications via their digital notification app (overlap of red and blue shaded circles; 0.2% of all cases). The effectiveness will be greater in the following scenarios. First, any case in the overlap of shaded red and unshaded blue circle (including persons who used at-home testing) can generate notifications via the app. Second, a larger shaded red circle reflects a higher proportion of the population actively using the digital notification app. Last, a larger unshaded black circle reflects a situation where more individuals can generate validation codes and receive exposure notifications. CICT, case investigation and contact tracing.

Greater use, increased compliance, or changes to digital notification system operations might increase its effectiveness (Figure 2). UK researchers assessing a similar app estimated that 167–349 cases were averted for every 1,000 notifications with a 28% adoption rate (*13*). Greater use appears achievable based on multiple reports indicating >17% of the population activated digital notification apps in 11 states and participation approached 50% in states where adoption was greatest (*6*,*7*). When we examined hypothetical scenarios in which 50% of the population actively used the app in Pennsylvania, all else remaining equal, we found that up to 3,995 cases could have been averted by digital notifications during the study period (Appendix). 

The potential increase in cases averted by digital notifications requires additional research and should consider other factors, such as alternative digital notification system operations. For example, effectiveness might be improved with automatic digital notification versus relying on case-patients to initiate contact notification after being interviewed. Some jurisdictions also started permitting users to self-report as COVID-19–positive and initiate digital notifications on the basis of at-home testing, which could improve both the number and timeliness of digital notifications (*14*). Although such gains are promising, they are moderated by the public’s compliance with digital notifications and technological limitations of Bluetooth signaling, leading to missed exposures and potentially false notifications.

Our findings suggest that the use of digital notification apps helped avert COVID-19 cases in Pennsylvania, although its effectiveness was limited by numerous factors, most notably limited use. The results also suggest opportunities exist to further examine and improve digital notification systems and their use during future outbreaks (Figure 2). Public health practitioners should explore ways to increase public participation in digital notification apps and to improve system efficiency by increasing the timeliness, coverage, and accuracy of digital notifications. 

Appendix Additional information on estimated cases averted by COVID-19 digital exposure notification, Pennsylvania, USA, November 8, 2020–January 2, 2021.
