# Supplementary material for: Estimated Cases Averted by COVID-19 Digital Exposure Notification, Pennsylvania, USA, November 8, 2020–January 2, 2021
Source: Emerg Infect Dis. 2023 Feb;29(2):426–30. doi: 10.3201/eid2902.220959 (PMC9881797; doi:10.3201/eid2902.220959)
Supplement: Appendix — Additional information on estimated cases averted by COVID-19 digital exposure notification, Pennsylvania, USA, November 8, 2020–January 2, 2021. [file 22-0959-Techapp-s1.pdf]

Article DOI: https://doi.org/10.3201/eid2902.220959

Estimated Cases Averted by COVID-19 Digital Exposure Notification, Pennsylvania, USA, November 8, 2020–January 2, 2021

Appendix

Additional Information and Assumptions

Users of the digital exposure notification applications (apps) were identified as contacts if they were within 6 feet for at least 15 minutes with another user who tested positive for COVID-19 within 14 days. Philadelphia County was excluded from the study because it was a separate health district with its own reporting and case investigation and contact tracing (CICT) programs. In total, 66 counties were included in the analysis with a total population of 11,217,925.

We used isolation and quarantine guidance from the US Centers for Disease Control and Prevention (CDC; https://www.cdc.gov/coronavirus/2019-ncov/your-health/isolation.html), as of this writing (April 1, 2022), which stated if you are sick or test positive for COVID-19 (i.e., a "case"), isolate for the recommended duration of the period even if you don't have symptoms; when you have been in close contact with someone who has COVID-19 (i.e., a "contact"), quarantine for the recommended duration of the period.

We defined the number of active digital notification app users as follows. For Android users, we used the total number of devices that had been turned on at least once in the past 30 days. For iOS users, we used the total number of devices with ≥1 session within 30 days of the selected day. During the study period, the median number of daily active users was 356,835, or 56% of users who downloaded the digital notification app.

Among 18 scenarios, we presented the lowest and highest estimates in the main text to show the range of results. The lowest value represented a scenario assuming only half of exposure notifications were sent to contacts not previously notified by standard CICT, and that 60% of interviewed cases and monitored contacts and 10% of notified contacts followed isolation and quarantine guidelines. The highest value represented a scenario assuming all

exposure notifications were sent to contacts not previously notified by standard CICT, and that 100% of interviewed cases and monitored contacts and 50% of notified contacts followed isolation and quarantine guidelines.

COVIDTracer Modeling Tool, Overview and Assumptions

COVIDTracer is a spreadsheet-based tool built by using a Susceptible-Exposed-Infectious-Recovered (SEIR) epidemiologic model to illustrate the spread of a pathogen, resultant disease, and the impact of interventions in a user-defined population (*1*). Readers can download the tool and enter input values of their choosing, exploring the impact of scenarios and assumptions beyond those covered in this manuscript. To model the clinical progression and transmission of disease using COVIDTracer, we used the following definitions and assumptions. A "case" was defined as a person who had been exposed, infected, and subsequently became infectious, regardless of the presence of clinical symptoms. We assumed that for the first 3 days after infection, cases do not infect others. During days 4–5 post-infection, cases are pre-symptomatic, but shed virus in amounts that infect others (*2–4*). During days 6–14, the infected person can be symptomatic and shedding virus, albeit during days 11–14 the risk of onward transmission is relatively low (the complete infectivity distribution is given in Appendix Table 1). We assumed that ≈40% of cases were asymptomatic during days 6–14 yet have a risk of onward transmission equal to 75% of symptomatic cases (Appendix Table 2) without vaccine or other nonpharmaceutical interventions (NPIs) (*4*). The model assumes homogeneous mixing among individuals and does not account for any age- or location-based heterogeneities in transmission (such as within and between households or schools), or variations in the effectiveness of vaccines and other NPIs over the study period. In addition, the tool employs a deterministic model which does not account for uncertainties around parameters. Users are encouraged to alter the default parameter values and perform sensitivity analysis to assess the impact of these assumptions; for example, the range of R_0 values (*5*,*6*) and age groups (Appendix Table 3).

We assumed that all notifications were sent to contacts who were truly exposed and the likelihood of becoming a case among digitally notified contacts was the same as those identified through standard CICT. As described in the main text, to account for the possibility that some notifications were sent to contacts that may have been identified through standard case

investigation, we assessed 2 scenarios by assuming 50% overlap between CICT and the digital notification app, and 0% overlap when calculating our cases and hospitalizations averted by the digital notification app. We assumed that contact notification speed for standard contact tracing and exposure notification was the same (i.e., 6 days post index case specimen collection) and that compliance among the 2 groups of contacts was the same (10% to 50%). Finally, we assumed that the effects of standard and digital notification programs remained constant over the 8-week study period.

Case Investigation and Contact Tracing Effectiveness

The effectiveness of CICT is determined by the proportion of cases and their infected contacts that are effectively isolated and quarantined, preventing further transmission in the susceptible population. The duration of quarantine and isolation is described in CDC guidance (*10*). We assumed that a proportion of confirmed cases are effectively isolated following case interviews. We further assumed that a proportion of contacts are quarantined upon either contact notification or through active monitoring.

We calculated the average proportion of cases and contacts isolated and quarantined by CICT as follows:

Step 1: We first calculated the proportion of cases that effectively isolated:

$$x * \left(\frac{No. cases\ that\ completed\ case\ interview}{Total\ number\ of\ cases}\right) = \text{Term A}$$

where x is the % of interviewed cases that isolated.

Step 2: We then calculated the proportion of contacts that effectively quarantined:

$$\%\ Contacts\ identified * (y * \%\ Contacts\ notified + x * \%\ Contacts\ monitored) = Term\ B$$

where:

% Contacts identified = $\frac{\#\ Contacts\ named\ by\ interviewed\ cases}{Total\ number\ of\ contacts}$ = Term B.1

% Contacts notified = $\frac{\#\ Contacts\ notified}{\#\ Contacts\ named\ by\ interviewed\ cases}$ = Term B.2

% Contacts monitored = $\frac{\#\ Contacts\ monitored}{\#\ Contacts\ named\ by\ interviewed\ cases}$ = Term B.2

y is % of cases among notified contacts (who are not monitored) that isolated, and x is the % of monitored contacts that isolated.

The "Total number of contacts" in Term B.1 was the expected total number of contacts generated by all cases. We estimated it by multiplying the reported total cases by the average number of contacts per case as follows:

$$Total\ Cases * \left(\frac{Total\ \#\ Contacts\ named\ by\ interviewed\ cases}{\#\ Cases\ that\ named\ at\ least\ 1\ contact}\right) = \text{Term B.1.1}$$

Step 3: To calculate the overall proportion of cases isolated by CICT among both interviewed cases and cases among their contacts we combined Terms A and B, weighting quarantined contacts by a multiplier *k*:

Average proportion of cases and contacts (that become cases) isolated =

$$\frac{(\%\ \text{Cases interviewed} * x) + \big(k * \%\ \text{Contacts identified} * (\%\ \text{Contacts monitored} * x + \%\ \text{Contacts notified} * y)\big)}{(1 + k)}$$

The multiplier *k* accounts for the expectation that the known case count represents just a fraction of the total secondary cases during our study period, since undetected infected contacts would have further infected additional individuals. Therefore, we used an approximation of the effective reproduction number (R_e) during our study period for the value of *k*: *k* = 1.2. If *k*>1 (i.e., the outbreak is growing), the proportion of contacts identified has a larger impact on the overall CICT effectiveness compared to the proportion of cases interviewed. Conversely, if *k*<1 (i.e., the outbreak is waning), the proportion of cases interviewed has a larger impact on the overall CICT effectiveness. R_e was fairly constant in Pennsylvania during our study period, varying only from 1.11–1.15 in our model. Therefore, a single value of *k* = 1.2 seemed a sufficient proxy over the short period of time we analyzed. However, we conducted a sensitivity analysis to examine the influence of alternate *k* values (see Sensitivity Analysis II: Varying the proportion of cases and contacts isolated/quarantined due to CICT).

In addition, reducing the time from case identification to effective isolation is critical for CICT to succeed. The longer that cases and contacts interact with the susceptible population, the greater the opportunity for onward transmission. In practice, cases with no known exposure are predominantly identified and isolated after symptom onset, and cases with known exposures (i.e.,

contacts that eventually become infected cases) can begin quarantine upon contact notification (even potentially before symptom onset). We assumed asymptomatic cases can only be identified and isolated if they are notified through CICT.

For our study, we assumed the proportions of cases with no known exposure and cases with known exposures were equal (i.e., 50/50 breakdown) because we did not have data on what prompted case identification. Therefore, the number of days to effective case isolation was determined by taking the average of days to effective isolation between case groups with known and unknown exposures. The time to effective case isolation for each of the two case groups was determined as follows.

For symptomatic cases with no known exposures (i.e., symptoms prompt identification), we assumed that cases experience a 5-day presymptomatic period (Appendix Table 2) and get tested the day after symptom onset (i.e., 6 days would have transpired since infection at the time of testing). We then obtained the number of days from exposure to result notification by adding the reported “Median days from specimen collection to case reporting to the health department.” We also assumed that confirmed cases begin isolation the day after their result notification (i.e., we added 1 to the total obtained above). Our assumptions regarding the “next-day” timing of testing and entry into isolation are based on symptoms and notifications beginning or occurring throughout the day, with a sizeable portion occurring sufficiently late enough in the day to prevent testing and entry into isolation the same evening. This assumption takes into account practical considerations, such as time needed to find a testing site and arrange an appointment, and for notified individuals to prepare to isolate (e.g., purchasing food or medications, setting up childcare, and handling work or other commitments).

For cases with known exposures (i.e., those who were notified they were a contact and eventually became a case), we assumed that contacts begin quarantine the day after receiving exposure notification from their health department (i.e., we added 1 to the sum above). The “next-day” timing of entry into quarantine is based on the same practical reasoning as cases needing time to prepare to isolate once notified (described above).

We then used the resultant sum from the procedure above to estimate the time in days from exposure to quarantine for contacts. Because we did not have information on when exposures actually occurred for contacts, we assumed that these contacts’ exposures occurred at

the midpoint of their potential exposure window in days. We identified the earliest date in this window as the first day of infectiousness among cases to which contacts were exposed. Based on our assumed 5-day presymptomatic period for symptomatic cases (described above), this was 2 days before the symptom onset date in cases exposing the contact. We identified the latest possible exposure as the date the cases exposing them were interviewed by the health department (because they began isolation the next day) (Appendix Figure 2).

The days between cases with known exposures becoming infected and their exposure notification can vary from what we assumed. For example, cases may take longer to become symptomatic, get tested the same day that they become symptomatic, or begin their isolation on the same day as their results notification. Similarly, contacts who become cases may be exposed earlier or later than we assumed and may make up a larger or smaller share of the case pool. Therefore, we performed a sensitivity analysis by varying the timing of isolation/quarantine by ±1 day from the estimated value (10 days). See Sensitivity Analysis III.

Using COVIDTracer Modeling Tool to Estimate Cases and Hospitalizations Averted

Step 1: Calculating CICT Effectiveness

We defined CICT effectiveness in terms of the coverage (percent of cases and contacts isolated and quarantined due to the CICT program) and the timeliness (number of days from exposure to isolation/quarantine). These values can be calculated by using the field-based data (e.g., percent of cases that completed case interview), and assumed values (e.g., public compliance to isolation and quarantine guidelines). See the section Case Investigation and Contact Tracing Effectiveness for more details.

Step 2: Adjusting for Effectiveness of Other NPIs

COVIDTracer tool allows users to attribute transmission reduction to either CICT or a combination of all other nonpharmaceutical interventions (NPIs), such as facemask wearing, social distancing, ban on large gatherings, and school and business closures. We first entered the calculated CICT effectiveness values into COVIDTracer. As the effectiveness of other NPIs is unknown, we toggled this value until the model-generated curve closely matched the reported case counts. The value that minimized the deviation (mean squared error, MSE) between the 2

curves was our estimated NPI effectiveness. We used the built-in "generalized reduced gradient non-linear" function in Excel (Microsoft, https://www.microsoft.com) to minimize the MSE.

Step 3: Simulating What Would Happen without EN

Next, we calculated the percent of cases and contacts isolated and quarantined, excluding the contribution of the digital notification app. This was done by excluding the proportion of contacts that were additionally identified and notified via the digital notification app. We then entered this value into the COVIDTracer tool, to simulate a hypothetical curve of what would have happened in the absence of digital notification. The difference between the reported case counts and this hypothetical curve was the estimated cases averted by digital notification.

The COVIDTracer tool is publicly available at https://www.cdc.gov/ncezid/dpei/resources/covid-tracer-Advanced-Special-edition.xlsm. The instructions provided in this Appendix can be used to replicate the analysis for any jurisdiction. See the section Instructions for Using COVIDTracer Modeling Tool to Estimate the Number of COVID-19 Cases and Hospitalizations Averted by Exposure Notification.

Isolation and Quarantine Compliance Scenarios: Sources and Details

A review of multiple cross-sectional population surveys in the United Kingdom suggests that 40%–45% of people who had COVID-19–like symptoms self-reported fully complying with isolation guidance during their infectious periods (*11*). Another survey in the United States found that 85% of respondents who had COVID-19–like symptoms or tested positive stayed home (according to CDC guidelines) except to get medical care (*12*). And a third survey, also in the United States, found that 93% of adults said they would definitely (73%) or probably (20%) quarantine themselves for at least 14 days if told to do so by a public health official because they had COVID-19 (i.e., they were confirmed cases, not just exposed contacts) (*13*).

Extended Results

Sensitivity Analysis I: Increased Digital Notification App Usage and Its Impact

Increasing the daily adoption rate for the digital notification app is one of the keys to maximizing the impact. We increased the currently reported 3.2% adoption rate (i.e., median number of daily active users during the 8-week period, divided by the population) up to 50%

(15.7 times higher than the baseline). Doubling the percent of app users may result in 4 times the number of notifications sent, because both twice the number of cases have the app and twice the number of contacts would be eligible to receive notifications. Similarly, increasing the adoption rate by 15.7 times may result in 247 times the number of notifications sent (n = 57,590).

Under the highest impact scenario, where we assume that all exposure notifications were sent to contacts that were not previously notified by standard CICT, and 50% of them complied with quarantine guidance, 3,995 additional cases and 98 hospitalizations would be averted by the app over the 8-week study period. However, this hypothetical scenario assumes that all other conditions remain equal (e.g., the operation of the EN system, % cases interviewed, % cases named at least one contact, compliance among notified users).

Sensitivity Analysis II: Varying the Proportion of Cases and Contacts Isolated/Quarantined Due to CICT

We used a value $k = 1.2$ to weight the proportion of contacts identified (thus quarantined) when calculating the overall proportion of cases and contacts isolated/quarantined by CICT (see Step 3 of Case Investigation and Contact Tracing Effectiveness section above). We approximated this value from the effective reproduction number (R_e) in Pennsylvania during our study period (model-estimated range 1.11–1.15). We, therefore, performed a sensitivity analysis in which we varied the weight (k) from 0.5 to 1.5. We provide results for this sensitivity analysis for the 2 scenarios that resulted in the lowest and highest EN impacts in our main analysis (Appendix Table 6).

Sensitivity Analysis III: Varying the Number of Days from Exposure to Isolation/Quarantined

In a previous study, we found that the time from infection of cases to their isolation and contacts' quarantine had the most influence on estimates of the number of cases and hospitalizations averted by CICT (*14*). Therefore, we varied the estimated number of days from exposure to isolation/quarantine by ± 1 day and estimated the impact of the app under each scenario. We provide results for this sensitivity analysis for the 2 scenarios that resulted in the lowest and highest impacts in the main analysis (Appendix Table 7).

Instructions for Using COVIDTracer Modeling Tool to Estimate the Number of COVID-19 Cases and Hospitalizations Averted by Digital Notification Apps

The COVIDTracer tool is available at https://www.cdc.gov/ncezid/dpei/resources/covid-tracer-Advanced-Special-edition.xlsm. These instructions will guide a user on how to use the Special Edition version of the COVIDTracer modeling tool to repeat the analysis described in this manuscript to estimate COVID-19 cases averted by case investigation and contact tracing activities. The Special Edition version is a modification of the publicly available tool on CDC's website that enables users to assess the impact of CICT before vaccine was widely available. Additional modifications would be required if you intend to explicitly account for vaccinated individuals (e.g., decreasing susceptible population over time, decreased risk of hospitalization among vaccinated individuals, etc.). COVIDTracer modeling tool uses the Windows 2010 or higher (Microsoft, https://www.microsoft.com) operating system and Excel (Microsoft Office 2013 or higher).

Before starting, complete the following:

1) Determine your 60-day study period. The first day of your study period is your "model start date." This "model start date" will be referenced later in these instructions. For example, if you are interested in estimating cases and hospitalizations averted by CICT during the 60-day period from January 1 to March 1, 2021, your "model start date" is January 1, 2021.
2) Obtain these data for the jurisdiction of interest:
    a. Total population
    b. Total cases as of the day before the model start date (In the example study period above, this is the total cases reported as of December 31, 2020.)
    c. Cases reported during the past 14 days (In the example study period above, this is the sum of cases reported from December 18 to 31, 2020.)
    d. The case trend during the past 14 days (*e.g.,* increasing, plateaued, decreasing)
    e. Daily (*i.e.,* incident) case counts for the 60-day study period
    f. The following case investigation and contact tracing program metrics. These metrics are meant to be representative of the 60-day study period. If you don't have such data for the entire study period, you may base these metrics on a shorter period (*e.g.,* 30 days or 4 weeks) from the model start date (and assume they are representative of the full 60 days):
        i. Number of days from exposure to case isolation and contact quarantine
        ii. Percent (%) of all cases successfully isolated and contacts quarantined
3) Open the COVIDTracer Advanced_SpecialEdition tool (downloaded from the link above)
    a. When opening the spreadsheet file, click the "Enable Macros" button for full functionality of the tool.

b. Enable Excel “Solver Add-In.” **Instructions**: in Excel, click on File → Options → Add-ins → select “Analysis ToolPak” → click “Go” (not the “Ok” button) → select checkbox for “Solver Add-In” and click “Ok.”

The Solver button, Solver will appear in the “Data” menu.

In worksheet A. Outbreak Details

Step 1: Enter the population for the jurisdiction of interest.

Enter the population of your jurisdiction

Total Population	1,000,000	*persons*

Step 2: Enter the model start date, the total number of COVID-19 cases in the jurisdiction until the day before the model start date, and the number of cases reported in the last 14 days within the jurisdiction.

Enter information about case counts in your jurisdiction

Start Date	1/1/2021	
Total Cases as of 12/31/2020	35,000	*cases*
Cases in the last 14 days (from 12/18/2020 to 12/31/2020)	5,000	*cases*

Note: These data inputs will only create curves for the purpose of calculating resources needs. They are not intended as, nor should be intepreted as, forecasts of future cases

Step 3: Set the pattern of daily cases over the past 14-day period selected in Step 3. The default is “Daily case counts are slowly increasing.” However, if daily case counts have been changing rapidly, remaining constant, or decreasing over the last 14 days, select from the pull-down menu the pattern that best matches the jurisdiction’s data.

The selection of the case trend in the past 14 days determines how reported cases are distributed over the 14 days prior to the model’s initiation date. Visually inspect the case trend and choose the most appropriate option. You can also run the model with different case trend patterns and pick one that yields the “best fit” (by repeating steps 3 to 6).

Enter estimates about the effectiveness of community interventions implemented through the present date

Are community interventions currently in place?	Yes		
Pattern of change in daily case counts over the last 14 days	Daily case counts are slowly increasing		How to choose?

In worksheet Case Counts

Step 4: Paste the jurisdiction’s daily case counts (i.e., incident cases) for the 60-day study period into the “Daily” column (column AH)

Cover	Case Counts	A. Outbreak Details	B. Impact of contact tracing

In Worksheet B. Impact of Contact Tracing

Step 5: Using your representative CICT program data, enter values for:

- Number of days after infection that case is isolated
- % of all cases successfully isolated and contacts traced and monitored (including both standard CICT and EN-notified contacts)

Set up the scenarios for contact tracing strategies

Contact Tracing Strategy Input

	No Contact Tracing	Continued Contact Tracing	Strategy 3 (Optional)
Number of days after infection that case is isolated	15	9	
% of all cases successfully isolated and contacts traced and monitored	0%	20.0%	
Strategy Trigger	Symptoms	Contact ID	
Contacts are identified and listed?	No	Yes	
Contacts follow-up occurs?	No	Yes	

**Successfully traced = the strategy worked as assumed and transmission to the next generation was prevented*

Step 6: Estimate the % reduction in transmission due to community interventions (shown in cell G28) by fitting COVIDTracer's simulated curve to your observed case curve. You will use the Solver Add-in to do this: The Solver Add-in finds an optimal solution for the % reduction in transmission due to community intervention by minimizing the mean squared error (a mathematical value describing the differences between both curves; shown in cell O32).

Instructions for Using the Solver

From the Excel menu tab, click "Data" and the "Solver" button, then follow the instructions described here to set up the parameters in the pop-up dialogue box (see screenshot below):

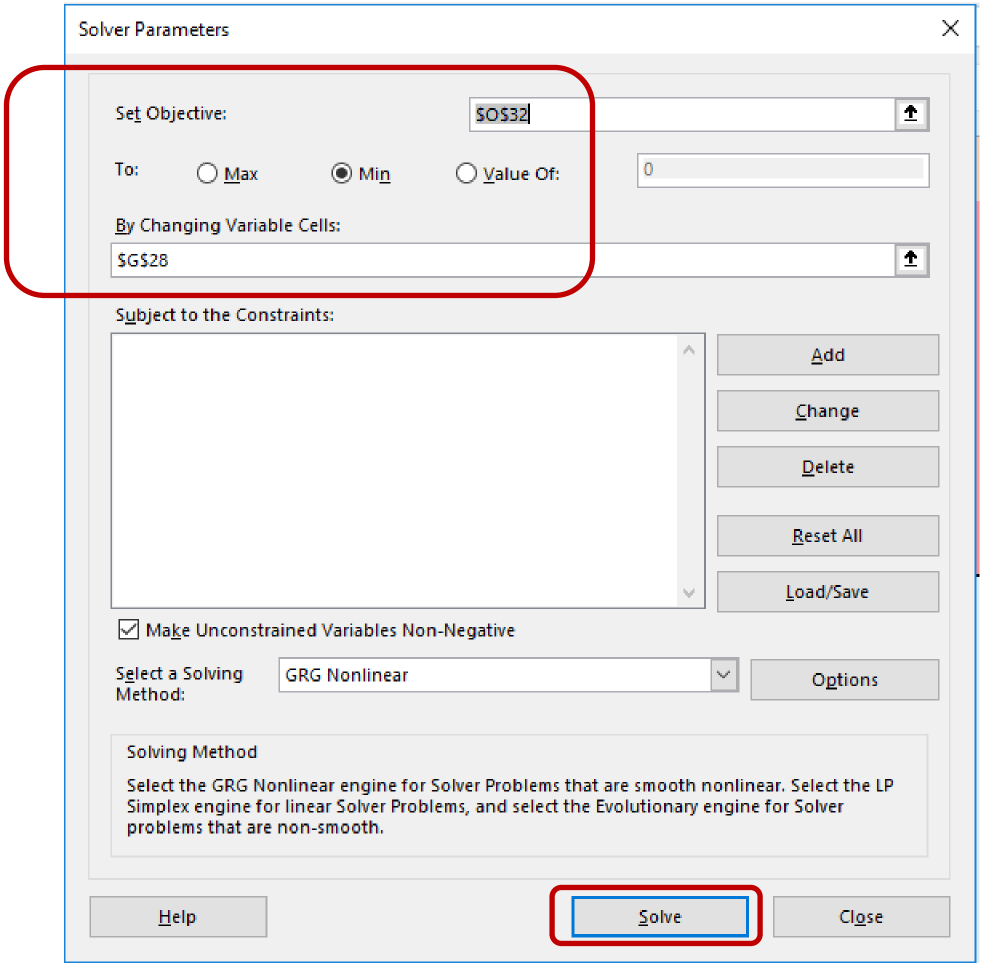

Set Objective: Set objective to cell “O32”, which is the mean squared error; To: Select “Min”.

By Changing Variable Cells: Enter G28 (This cell refers the Solver to the “Estimated % reduction in transmission due to continued community interventions.”)

Select a Solving Method: For simplicity, we recommend selecting “GRG Nonlinear” from the drop-down menu.

Click “Solve” button. Then the Excel Solver function will automatically find the optimal value (estimated % reduction in transmission due to continued community intervention) and populate the value in cell G28. The figure below shows a fitted curve (solid line) generated by COVIDTracer after Step 6, that minimizes deviation from the reported case counts (dashed line).

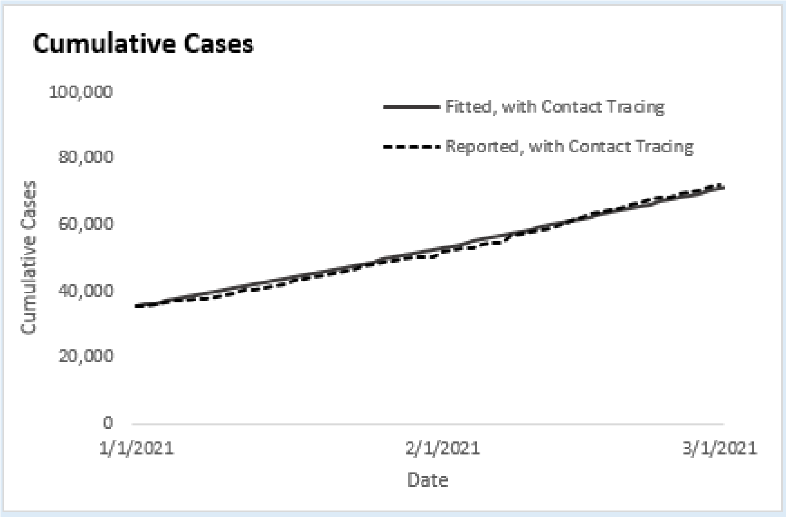

Example Figure. Fitted curve using COVIDTracer modeling tool.

In Worksheet Results – Cases Averted

Step 7. Users can find the % reduction in transmission due to CICT, and those that are attributable to all other interventions. The estimated number of cases and hospitalizations averted by CICT is also provided on this page. This is the combined impact of standard CICT and exposure notification (EN).

Transmission Fraction

Transmission Reduction from Contact Tracing	4.5%
Transmission Reduction from All Other Interventions	54.7%
Remaining Transmission*	43.3%

*Calculated as follows: (1-reduction from CT) * (1-reduction from other interventions)

Cases Averted, 60 days

Cases Averted by Contact Tracing	8,937
Cases Averted per 100,000 population	894
% of Additional Cases Averted by Contact Tracing**	19.7%

**Additional cases averted by contact tracing out of every 100 remaining cases after accounting for the impact of all other interventions (e.g., vaccination, facemask policies, social distancing).

Hospitalizations Averted, 60 days

Hospitalizations Averted by Contact Tracing	220
Hospitalizations averted per 100,000 population	22
% of Additional Hospitalizations Averted by Contact Tracing***	19.7%

***Additional hospitalizations averted by contact tracing out of every 100 remaining hospitalizations after accounting for the impact of all other interventions.

Go back to Worksheet B. Impact of Contact Tracing

Step 8: Replace the % of all cases successfully isolated and contacts traced and monitored, with the value excluding the contributions of EN.

Move onto Worksheet Results – Cases Averted

Step 9: Now the results on this page will show the impact of standard CICT alone, based on a hypothetical scenario of what would've happened without digital notification. If, for example, the combined impact of standard CICT and EN was 8,937 cases being averted (from Step 7), and the standard CICT alone averted 8,900 cases (from Step 9), then the difference (37 in this example) is the additional cases being averted by EN.

References

1. Adhikari BB, Arifkhanova A, Coronado F, Fischer LS, Greening B, Jeon S, et al. COVIDTracer Advanced: a planning tool to illustrate the resources needed to conduct contact tracing and monitoring of coronavirus disease 2019 (COVID-19) cases and the potential impact of community interventions and contact tracing efforts on the spread of COVID-19 [cited 2022 May 1]. https://www.cdc.gov/coronavirus/2019-ncov/downloads/php/contact-tracing/AdvancedCOVIDTracerManual.pdf

2. He X, Lau EHY, Wu P, Deng X, Wang J, Hao X, et al. Temporal dynamics in viral shedding and transmissibility of COVID-19. Nat Med. 2020;26:672–5. PubMed https://doi.org/10.1038/s41591-020-0869-5

3. Ferretti L, Wymant C, Kendall M, Zhao L, Nurtay A, Abeler-Dörner L, et al. Quantifying SARS-CoV-2 transmission suggests epidemic control with digital contact tracing. Science. 2020;368:eabb6936. PubMed https://doi.org/10.1126/science.abb6936

4. US Centers for Disease Control and Prevention. COVID-19 pandemic planning scenarios; 2021 Mar 19 [cited 2022 May 1]. https://www.cdc.gov/coronavirus/2019-ncov/hcp/planning-scenarios.html

5. Liu Y, Rocklöv J. The reproductive number of the Delta variant of SARS-CoV-2 is far higher compared to the ancestral SARS-CoV-2 virus. J Travel Med. 2021;28:taab124. PubMed https://doi.org/10.1093/jtm/taab124

6. Ge Y, Martinez L, Sun S, Chen Z, Zhang F, Li F, et al. COVID-19 transmission dynamics among close contacts of index patients with COVID-19: a population-based cohort study in Zhejiang Province, China. JAMA Intern Med. 2021;181:1343–50. PubMed https://doi.org/10.1001/jamainternmed.2021.4686

7. US Centers for Disease Control and Prevention. COVID data tracker [cited 2022 May 1]. https://covid.cdc.gov/covid-data-tracker

8. Bialek S, Boundy E, Bowen V, Chow N, Cohn A, Dowling N, et al.; CDC COVID-19 Response Team. Severe outcomes among patients with coronavirus disease 2019 (COVID-19)—United States,

February 12–March 16, 2020. MMWR Morb Mortal Wkly Rep. 2020;69:343–6. PubMed https://doi.org/10.15585/mmwr.mm6912e2

9. Wu SL, Mertens AN, Crider YS, Nguyen A, Pokpongkiat NN, Djajadi S, et al. Substantial underestimation of SARS-CoV-2 infection in the United States. Nat Commun. 2020;11:4507. PubMed https://doi.org/10.1038/s41467-020-18272-4

10. US Centers for Disease Control and Prevention. COVID-19 quarantine and isolation guidance [cited 2022 May 1]. https://www.cdc.gov/coronavirus/2019-ncov/your-health/quarantine-isolation.html

11. Smith LE, Potts HWW, Amlôt R, Fear NT, Michie S, Rubin GJ. Adherence to the test, trace, and isolate system in the UK: results from 37 nationally representative surveys. BMJ. 2021;372:n608. PubMed https://doi.org/10.1136/bmj.n608

12. Park CL, Russell BS, Fendrich M, Finkelstein-Fox L, Hutchison M, Becker J. Americans' COVID-19 stress, coping, and adherence to CDC guidelines. J Gen Intern Med. 2020;35:2296–303. PubMed https://doi.org/10.1007/s11606-020-05898-9

13. Pew Research Center. The challenges of contact tracing as U.S. battles COVID-19 [cited 2022 May 1]. https://www.pewresearch.org/internet/2020/10/30/the-challenges-of-contact-tracing-as-u-s-battles-covid-19

14. Jeon S, Rainisch G, Lash RR, Moonan PK, Oeltmann JE Jr, Greening B Jr, et al.; Contact Tracing Impact Group. Estimates of cases and hospitalizations averted by COVID-19 case investigation and contact tracing in 14 health jurisdictions in the United States. J Public Health Manag Pract. 2022;28:16–24. PubMed https://doi.org/10.1097/PHH.0000000000001420

Appendix Table 1. Daily percentage risk for transmission by infectiousness state and clinical symptoms in a study of estimated cases averted by COVID-19 digital exposure notification, Pennsylvania, USA, November 8, 2020–January 2, 2021*

Days post infection	% Daily risk for onward transmission†	Infected person's state
1	0.00	Infected, not yet infectious
2	0.00	
3	0.00	
4	16.78	Infectious, presymptomatic
5	18.03	
6	17.07	Infectious, symptomatic
7	14.52	
8	11.27	
9	8.10	
10	5.48	
11	3.55	
12	2.26	
13	1.46	
14	1.48	
Total	100	

*Sources include He et al. (*2*) and Ferretti et al. (*3*). See also COVIDTracer modeling tool manual (*1*).
†Percentages show when onward transmission might occur by the day of infectiousness.

Appendix Table 2. Epidemiologic parameters, values, and sources used in a study of estimated cases averted by COVID-19 digital exposure notification, Pennsylvania, USA, November 8, 2020–January 2, 2021

Parameter	Default value	Source
Infected but not yet infectious period	3 d	CDC COVID-19 Pandemic Planning Scenarios (*4*)
Presymptomatic and contagious (infectious) period	2 d	He et al. (*2*), Ferretti et al. (*3*)
Symptomatic and contagious (infectious) period	9 d	He et al. (*2*), Ferretti et al. (*3*)
Basic reproduction number (R_0)	2.5	CDC COVID-19 Pandemic Planning Scenarios (*4*)
% Asymptomatic cases	40	CDC COVID-19 Pandemic Planning Scenarios (*4*)
% Infectiousness of asymptomatic cases relative to symptomatic cases	75	CDC COVID-19 Pandemic Planning Scenarios (*4*)

Appendix Table 3. Default values in COVIDTracer and sources for assumed proportion of cases by age group and infection-to-hospitalization rate in a study of estimated cases averted by COVID-19 digital exposure notification, Pennsylvania, USA, November 8, 2020–January 2, 2021*

Age group, y	% Total cases	Source	% Cases admitted to hospital care	Source
0–17	15	CDC COVID Data Tracker (*7*)	0.21	CDC COVID-19 Response
18–64	55		2.17	Team (*8*), Wu et al. (*9*)
>65	30		4.12	

*Assumptions derived September 2020 by using sources available at that time. CDC, US Centers for Disease Control and Prevention

Appendix Table 4. Estimated number of cases and hospitalizations averted by standard contact tracing and exposure notification in a study of estimated cases averted by COVID-19 digital exposure notification, Pennsylvania, USA, November 8, 2020–January 2, 2021*

	No. averted		
Isolation and quarantine compliance	Standard CICT	EN	No. averted by EN/1,000 notifications
60% Cases and monitored contacts			
10% Notified contacts			
Cases	10,168	3.20	13.7
Hospitalizations	250	0.08	0.3
30% Notified contacts			
Cases	10,180	9.60	41.2
Hospitalizations	250	0.24	1.0
50% Notified contacts			
Cases	10,192	15.99	68.6
Hospitalizations	250	0.39	1.7
80% Cases and monitored contacts			
10% Notified contacts			
Cases	13,628	3.21	13.8
Hospitalizations	335	0.08	0.3
30% Notified contacts			
Cases	13,641	9.62	41.3
Hospitalizations	335	0.24	1.0
50% Notified contacts			
Cases	13,653	16.03	68.8
Hospitalizations	335	0.39	1.7
100% Cases and monitored contacts			
10% Notified contacts			
Cases	17,126	3.21	13.8
Hospitalizations	421	0.08	0.3
30% Notified contacts			
Cases	17,138	9.64	41.4
Hospitalizations	421	0.24	1.0
50% Notified contacts			
Cases	17,151	16.07	68.9
Hospitalizations	421	0.39	1.7

*We assumed all exposure notifications (n = 233) were sent to contacts not previously notified by standard CICT. The analysis excludes Philadelphia County. We assumed 60%–100% of interviewed cases, 60%–100% of monitored contacts, and 10%–50% of notified but not monitored contacts fully adhered to isolation and quarantine guidance. The table provides the number of cases and hospitalizations averted by standard CICT and EN under each compliance scenario, when all exposure notifications were assumed to be sent to contacts not notified by the standard program. We assumed all exposure notifications were sent to true contacts. CICT, case investigation and contact tracing; EN, exposure notification.

Appendix Table 5. Estimated number of cases and hospitalizations averted by standard contact tracing and exposure notification in a study of estimated cases averted by COVID-19 digital exposure notification, Pennsylvania, USA, November 8, 2020–January 2, 2021*

	No. averted		
Isolation and quarantine compliance	Standard CICT	EN	No. averted by EN/1,000 notifications
60% Cases and monitored contacts			
10% Notified contacts			
Cases	10,168	1.60	6.9
Hospitalizations	250	0.04	0.2
30% Notified contacts			
Cases	10,180	4.80	20.6
Hospitalizations	250	0.12	0.5
50% Notified contacts			
Cases	10,192	8.00	34.3
Hospitalizations	250	0.20	0.8
80% Cases and monitored contacts			
10% Notified contacts			
Cases	13,628	1.60	6.9
Hospitalizations	335	0.04	0.2
30% Notified contacts			
Cases	13,640	4.81	20.6
Hospitalizations	335	0.12	0.5
50% Notified contacts			
Cases	13,652	8.02	34.4
Hospitalizations	335	0.20	0.8
100% Cases and monitored contacts			
10% Notified contacts			
Cases	17,126	1.61	6.9
Hospitalizations	421	0.04	0.2
30% Notified contacts			
Cases	17,138	4.82	20.7
Hospitalizations	421	0.12	0.5
50% Notified contacts			
Cases	17,150	8.03	34.5
Hospitalizations	421	0.20	0.8

*We assumed 50% of exposure notifications (n = 117) were sent to contacts not previously notified by standard CICT. The analysis excludes Philadelphia County. We assumed 60%–100% of interviewed cases, 60%–100% of monitored contacts, and 10%–50% of notified but not monitored contacts fully adhered to isolation and quarantine guidance. The table provides the number of cases and hospitalizations averted by standard CICT and EN under each compliance scenario, when all exposure notifications were assumed to be sent to contacts not notified by the standard program. We assumed all exposure notifications were sent to true contacts. CICT, case investigation and contact tracing; EN, exposure notification.

Appendix Table 6. Sensitivity analysis results varying proportion of isolated cases and contacts in a model used to estimate cases averted by COVID-19 digital exposure notification, Pennsylvania, USA, November 8, 2020–January 2, 2021*

	CICT effectiveness	Estimated impact of EN†	
Weight, κ	% Cases and contacts isolated	Lowest impact scenario‡	Highest impact scenario§
0.5	9.0–15.0	8.4	42.3
1.2, baseline	7.0–11.7	6.9	68.9
1.5	6.5–10.8	15.1	75.8

*CICT, case investigation and contact tracing; EN, exposure notification.
†Cases averted per 1,000 notifications.
‡Assuming 50% of notifications sent to contacts not previously identified by standard CICT. It further assumes that 60% of interviewed cases and monitored contacts followed the guidelines, and 10% of notified contacts followed quarantine guidelines.
§Assuming all notifications were sent to contacts not previously identified by standard CICT. It further assumes that 80% of interviewed cases and monitored contacts followed the guidelines, and 50% of notified contacts followed quarantine guidelines.

Appendix Table 7. Sensitivity analysis results varying days from exposure to isolation quarantine in a model used to estimate cases averted by COVID-19 digital exposure notification, Pennsylvania, USA, November 8, 2020–January 2, 2021*

No. days from exposure to isolation/quarantine	Estimated impact of EN†	
	Lowest impact scenario‡	Lowest impact scenario§
9	11.7	117.5
10, baseline	6.9	68.9
11	3.9	39.2

*CICT, case investigation and contact tracing; EN, exposure notification.
†Cases averted per 1,000 notifications.
‡Assuming 50% of notifications sent to contacts not previously identified by standard CICT. It further assumes that 60% of interviewed cases and monitored contacts followed the guidelines, and 10% of notified contacts followed quarantine guidelines.
§Assuming all notifications were sent to contacts not previously identified by standard CICT. It further assumes that 80% of interviewed cases and monitored contacts followed the guidelines, and 50% of notified contacts followed quarantine guidelines.

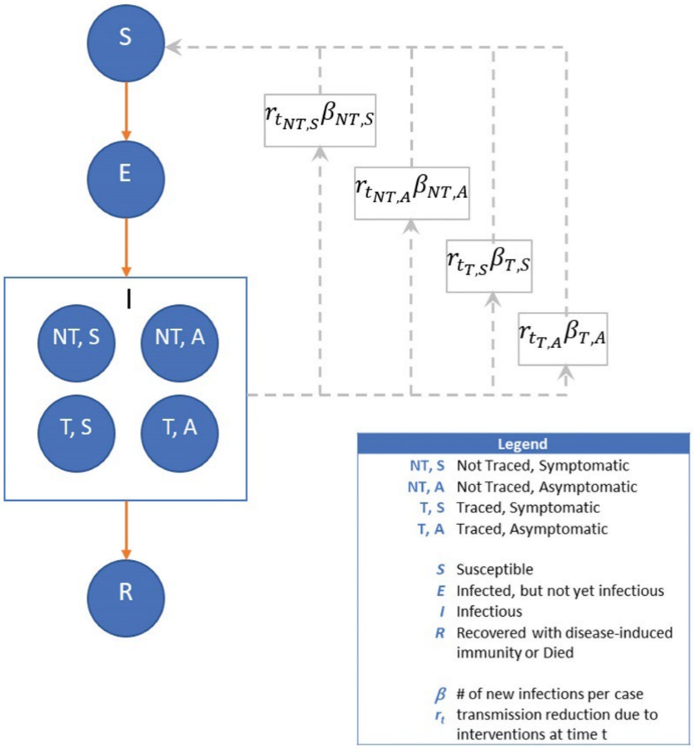

Appendix Figure 1. COVIDTracer model structure used to estimate cases averted by COVID-19 digital exposure notification, Pennsylvania, USA, November 8, 2020–January 2, 2021. The model consists of cases who are either Susceptible (*S*), Infected but not yet Infectious (*E*), Infectious (*I*), Recovered or Dead (*R*). Cases can move between these compartments as indicated by the orange arrows. The model tracks the number of cases moving between these categories every day of the outbreak. The rate of new infections is influenced by the number of cases in the *I* category (depicted by the light gray dashed lines). There are 4 types of *I* cases: symptomatic or asymptomatic persons who adhere to isolation guidelines because they were engaged by their health departments via case investigation and contact tracing efforts (CICT); and symptomatic or asymptomatic persons who do not participate in CICT efforts. The overall risk for onward transmission to the *S* population is dependent upon both the distribution of cases among these 4 infectious categories on each day, and any reductions in transmission associated with a jurisdiction's implementation of CICT, and vaccine and other, nonpharmaceutical interventions.

	Day 1	2	3	4	5	6	7	8	9	10	11	12	Day 13	Day 14	Days from Exposure to Isolation
**Index Case**	Exposed			Contagious Period Begins		Symptom Onset	Tested					Result Notification & Case Interview	Begin Isolation		12
**Contacts** (Earliest possible exposure)				Exposed									Exposure Notification	Begin Quarantine	10
**Contacts** (Latest possible exposure)												Exposed	Exposure Notification	Begin Quarantine	2

Appendix Figure 2. Timeline of COVID-19 case isolation and quarantine of contacts in a model used to estimate cases averted by COVID-19 digital exposure notification, Pennsylvania, USA, November 8, 2020–January 2, 2021. We assumed a 5-day presymptomatic period and that confirmed cases got tested the day after symptom onset. The state of Pennsylvania reported 5 days from specimen collection to the case interview and 6 days for contact notification. The index case (symptomatic case with no known exposure) began showing symptoms on day 6 post-infection, got tested on day 7, and was notified of the test result on day 12. The case's contacts (cases with known exposure) were exposed sometime between days 4–12 and notified of their exposure on day 13. Therefore, the index case began isolation on day 13 and contacts went into quarantine on day 14 (based on our assumptions above). To calculate the days from contacts' exposure to their quarantine, we took the average of the maximum days a contact was infected (10 days, based on the earliest possible exposure) and the fewest days the contact could be infected (2 days, based on the latest possible exposure), and weighted each day span by the case's infectiousness on each of the possible exposure days. The result is 7.3 days in this example, meaning the contact had been exposed for 7.3 days upon initiating quarantine. We then took the average between 12 days (index case) and 7.3 days (contacts) as the number of days from exposure to isolation (for both cases and contacts), which was 10 days. This final value (10 days) represents one of the key case investigation and contact tracing performance metrics, the number of days from exposure to isolation/quarantine.
